# Effects of metastable resistance training with strength and balance requirements compared to traditional resistance and balance training on cognitive performance in older adults: a randomized controlled trial

**DOI:** 10.1186/s12877-025-06438-y

**Published:** 2025-10-15

**Authors:** Lisa Claußen, Julian Groß, Armin Kibele

**Affiliations:** Institute of Sports and Sport Science, University of Kassel, Kassel, Germany

**Keywords:** Exercise-cognition interaction, Instability, Machine-based resistance training, Free-weight resistance training, Chronic effects, Brain health, Aging

## Abstract

**Background:**

While aerobic, resistance, and balance training are commonly used to counteract age-related declines in cognitive and physical functions, evidence for cognitive benefits of metabolic training (i.e., aerobic, resistance) remains inconsistent. In contrast, motor training (i.e., balance, coordination) involving higher task complexity may enhance cognition by engaging brain regions associated with cognitive control processes. Resistance training on unstable devices, also referred to as metastable resistance training (MRT), has been reported to increase metabolic, coordinative, and cognitive demands during exercise, as well as to improve cognitive performance in older adults. This study examined the effect of MRT on cognitive performance compared to traditionally recommended resistance training (T-RT) and balance training (BT). We hypothesized that MRT specifically improves cognitive task performance requiring perceptual processing and attention.

**Methods:**

Eighty-three healthy older adults (mean age 70.5 ± 4.5 years) were matched into three groups which were randomly assigned to either MRT, BT or T-RT programs. Each group trained twice a week for 10 weeks. Cognitive functions were assessed using four tasks targeting working memory, inhibitory control, cognitive flexibility, and perceptual processing. Linear mixed-effects models were applied to examine the effect of MRT on cognitive performance in contrast to BT and T-RT.

**Results:**

A significant time-by-group interaction was observed for inhibitory control when contrasting MRT with BT, *t*(80) = 3.56, *p* < 0.001, *β* = 0.42, 95% CI [0.19, 0.65], indicating improved response inhibition following MRT. Additionally, perceptual processing was significantly enhanced when comparing MRT with BT for both reaction time, *t*(79) = 2.35, *p* = 0.020, *β* = 0.19, 95% CI [0.03, 0.35], and accuracy, *t*(79) = -2.69, *p* = 0.009, *β* = -0.26, 95% CI [-0.45, -0.07].

**Conclusions:**

In contrast to BT, MRT appears to selectively enhance cognitive functions requiring inhibitory control and perceptual processing in older adults. Consequently, metabolic demands associated with MRT may offer additional cognitive benefits beyond the coordinative demands offered by traditional balance training.

**Clinical trial number:**

This trial, DRKS00030394, was registered in the German Clinical Trials Register on 16/08/2023.

## Background

Physical and cognitive functions develop throughout life and decline with age [1–3]. In addition to general cognitive slowing and the deterioration of information processing [4, 5], the executive functions are arguably the cognitive functions most impacted by the aging process [6, 7]. The term executive function involves top-down cognitive control processes that are important for effective, goal-directed actions, particularly in situations where action must be taken contrary to habitual patterns, when problem-solving or error correction is required, or when newly learned movements must be executed [8, 9]. In general, they are essential for maintaining mental and physical health [10] as well as for the management of activities of daily living [11, 12]. In an aging population, a decline in executive functions is associated with an increased risk of dementia, impaired balance and postural control, and thus an increased risk of falls [13, 14]. Falls pose a significant threat to the lives of older adults, with serious consequences associated with injuries and potential loss of life independence [15, 16].

Regular physical and motor training are both recommended by the American College of Sports Medicine to reduce the risk of falls and maintain independence in old age [17]. Physical training, such as aerobic and resistance training, can be described as intensity-based training with a repetitive and automatic character to improve physical fitness (i.e., endurance, strength). Contrarily, motor training is characterized by complex motor control tasks and low metabolic demands, aimed at improving motor fitness (i.e., balance, coordination) [18]. Studies have shown that both types of training can have a positive impact not only on physical performance, but also on cognitive functioning in older adults [18, 19]. Physical training and fitness, in general, are positively associated with brain health and structural and functional plasticity [20, 21]. Resistance training specifically appears to maintain and enhance cognitive function and performance in older adults by promoting angiogenesis and neuroplasticity, which are related to the production of neurotrophic factors, including Insulin-like Growth Factor 1 (IGF-1), Brain-Derived Neurotrophic Factor (BDNF), and Vascular Endothelial Growth Factor (VEFG) [22]. These possible underlying mechanisms of physical training on cognition are summarized as the *intensity pathway* [23, 24].

Nevertheless, contradictory results of intervention studies and meta-analyses [25, 26], along with insufficient evidence on the mechanisms underlying the relationship between resistance training and cognitive function, have led to ongoing debate. Critics argue that resistance training’s repetitive and automatic nature offers minimal cognitive engagement, insufficient for enhancing executive functions [27, 28]. Supporting this argument, theoretical assumptions postulate that novel and complex motor tasks require focused attention for successful completion, thereby activating and stimulating brain regions associated with cognitive control processes which are also crucial for performance in executive function tasks [29]. Accordingly, the term *complexity pathway* is used to describe the underlying mechanism through which motor training affects cognition [23, 24].

In line with these assumptions, behavioral and neuroimaging data from studies comparing physical with motor training interventions demonstrate both comparable and distinct effects on cognition [30–32]. Specifically, a twelve-month coordination training program proved to be as beneficial as aerobic training in enhancing executive performance, yet more effective in improving perceptual speed in older adults. Neuroimaging data further revealed that coordination training enhanced cognitive processing efficiency in task-related brain areas, comparable to that observed following cardiovascular training, but also enhanced processing and integration of visuospatial information [32]. To further expand research on the exercise-cognition interaction, randomized controlled trials are needed that modulate type of exercise and task complexity alongside the quantitative exercise parameters such as frequency, intensity, and duration [23, 24, 33]. The present study addresses this proposition by comparing the effects of singular and combined resistance and balance training programs with distinct physical and motor demands on cognitive and physical performance in older adults.

Metastable resistance training (MRT), which involves free-weight resistance training on unstable surfaces, integrates both physical and motor training aspects by simultaneously demanding strength and balance. Recent research highlights that MRT can significantly enhance muscle strength, balance, and mobility in older adults when implemented progressively and under appropriate supervision [34, 35]. Current studies on the acute effects of MRT demonstrate that exercising on unstable surfaces (e.g., squats) increases metabolic demands [36], alters coordinative requirements [37, 38], and necessitates greater attentional resources [39]. Furthermore, engaging in MRT is associated with higher task complexity, leading to significantly more cognitive activation than exercises performed on stable surfaces [40]. Moreover, the research by Eckardt et al. [41] showed that engaging in MRT for a period of 10 weeks results in notable improvements in cognitive performance and executive functions - such as processing speed, working memory, and inhibitory control - compared to traditional machine-based resistance training (T-RT). The positive outcomes of MRT are attributed to its physically and cognitively demanding nature [41] and may be mediated by increased levels of neurotrophic factors as well as the recruitment of motor skill learning processes. In a recent study by Cavalcante et al. [42] the effectiveness of MRT and T-RT was examined among older adults with cognitive impairments. Although no significant improvements were witnessed overall, MRT showed superior enhancements in global cognition and memory performance in comparison to T-RT [42].

Presently, the majority of research investigating the efficacy of MRT in boosting cognitive performance has predominantly compared it to other physically and metabolically intense training regimes. However, there has not yet been a comparison with exclusively motor-based training, such as balance training. Therefore, the purpose of this study was to examine the combined effect of metabolic and coordinative demands on healthy older adults’ cognitive and physical performance by comparing MRT with traditional balance and resistance training. Employing a range of established cognitive and executive function tasks, the study was designed to evaluate the effect of MRT on core executive functions (working memory, inhibitory control, cognitive flexibility) [10, 43] as well as visuospatial processing [44]. We hypothesized that MRT shows higher effects on manipulation of information in a working memory task [45], on inhibitory control [41], and perceptual processing [32] compared to traditional balance and resistance training due to its coordinative and metabolic demands.

## Methods

### Study design

This study is part of a registered randomized controlled trial with three parallel groups that investigated the effects of three exercise modalities with differing strength and balance requirements on physical and cognitive resources of older adults (DRKS00030394 on 16/08/2023). The participants were unaware of the study’s goals and the examiners were blinded to the participants’ assignments. The local ethics committee of the University of Kassel granted approval (E05202301). The study adhered to the ethical standards of the current Declaration of Helsinki (WMA, October 2013). All participants provided written informed consent prior to participating in the study.

### Sample size calculation

We conducted an a priori sample size estimation using G*Power 3.1.9.7. We used the effect size (*d* = 0.42) obtained for the Stroop Score by Eckardt et al. [41] and ran our power analysis for a repeated measures analysis of variance with within-between interaction, with three groups and two measurements as the independent variables. We set the test power at 0.80, with a Type I error rate of α = 0.05. The power analysis revealed that a total sample size of 222 was required when applying the effect size specification as in Cohen [46]. However, due to financial and time constraints, we had to limit the sample size to 96.

### Participants

**Fig. 1:**
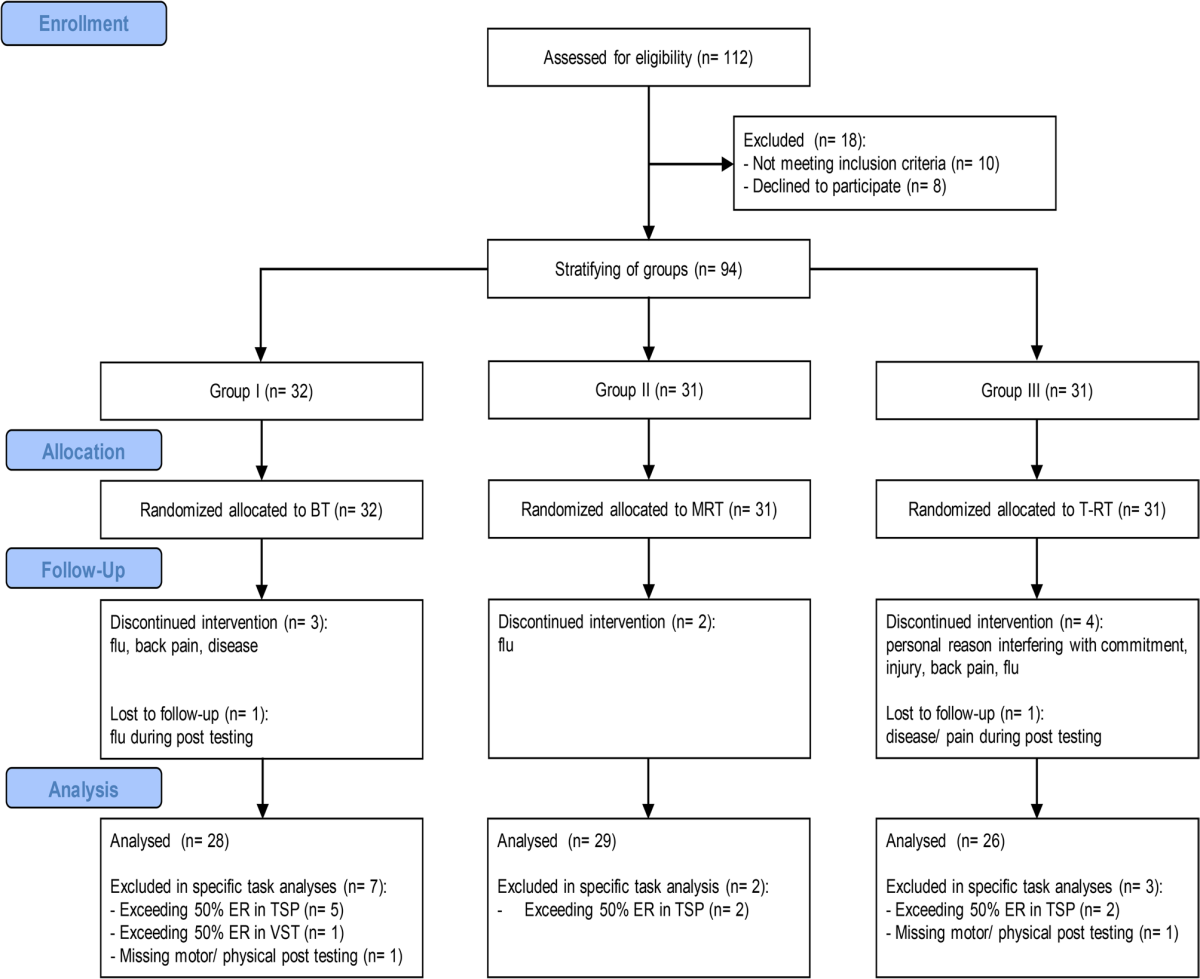
CONSORT Flowchart of Participants Note. BT = balance training; ER = error rate; MRT = metastable resistance training; T-RT = traditional resistance training; TSP = task-switching paradigm; VST = visual search task

A total of 112 participants, aged between 65 and 83 years, were recruited via a public advertisement in a local newspaper. Before being invited to the lab for baseline testing, socio-demographic data (e.g., age, gender, education), health status (e.g., medical conditions), health behavior (e.g., physical activity level), and psychosocial variables (e.g., depression, self-efficacy) were collected online through a LimeSurvey questionnaire (version 3.28.73 + 230906, LimeSurvey GmbH, Hamburg, Germany) or by a paper-and-pencil questionnaire. The inclusion criteria included the ability to walk independently without the use of a walking aid, normal or corrected-to-normal vision, and the absence of a neurological disease (e.g., Parkinson’s disease). All information regarding inclusion and exclusion criteria was self-reported. The physical activity level was assessed using the German Physical Activity Questionnaire for Population 50+ (PAQ-50+) [47], depressive symptoms were analyzed using the short form of the Geriatric Depression Scale (GDS-15) [48], and fall-related self-efficacy was measured using the German translation of the Activities-specific Balance Confidence Scale (ABC-D) [49]. Global cognitive performance was evaluated using the Montreal Cognitive Assessment (MoCA) [50], a screening instrument for mild cognitive impairment, on the first day of testing by trained and certified examiners. Participants were excluded if they scored below 23 on the MoCA [51] or above 5 on the GDS-15 [52] to account for the potential influence of cognitive impairment and mental illness. 83 participants completed the exercise intervention, with two individuals missing the motor fitness assessment of the post-testing sessions. Figure 1 shows the CONSORT flowchart illustrating the number of participants in the intervention groups at each stage of the study. A sensitivity analysis on the actual sample size of 83 participants, with an α-level of 0.05 and a power of *β* = 0.8, showed that only medium-to-large effects (*d* = 0.7) can be detected with this sample size.

### Stratification and randomisation

Participants were matched on sociodemographic and behavioral characteristics to increase comparability between groups and reduce confounding. These characteristics included age, gender, years of education, amount of medication, current resistance training habits, and fall-related self-efficacy. In detail, we assigned numerical codes to each category (e.g., gender: 1 = male, 2 = female; age: 1 = 65–69 years, 2 = 70–74 years, 3 = 75–79 years, 4 = > 80 years). We then matched individuals with similar characteristics, such as age group, gender group, etc., to form triplets. The order of the traits was as follows: (1) gender, (2) age, (3) education (1 = 12 years of education or less, 2 = more than 12 years of education), (4) current resistance training practice (1 = never, 2 = at least once in a month, 3 = at least once a week), (5) designation as a sports person (0 = no, 1 = yes), (6) mean score of the ABC-D (1 = ≤ 80%, 2 = 81–90%, 3 = > 90%), (7) amount of medication (1 = 0–1, 2 = 2–5, 3 = > 5). Each person in the triplet was assigned to a (training) group, which were then filled with individuals whose characteristics did not exactly match those of the others. The final grouping was checked for gender balance and similar mean age. Finally, a blinded research assistant randomly assigned the matched groups to one of three training modalities using sealed opaque envelopes: balance training (BT), metastable free-weight resistance training (MRT), and traditional machine-based resistance training (T-RT).

### Assessments

Pre-intervention (baseline) and post-intervention measurements of cognitive, physical and motor performance were conducted in the movement science laboratory rooms of the University of Kassel, Germany, by raters blinded to participant assignment.

### Primary measures: executive function and perceptual processing

To assess executive functions, the study focused on the following components: working memory, inhibitory control, and cognitive flexibility [10, 43]. Additionally, perceptual processing was assessed to differentiate the effects of training modalities with varying metabolic and coordinative demands. All cognitive tasks were conducted on a computer. Participants were seated at a distance of roughly 60 cm from a 24-inch LCD monitor (ASUS VG248QE, 1980 × 1080 pixels, 60 Hz). The order of tasks was randomly assigned to each participant prior to the pre-session and maintained throughout the post-session (www.randomizer.org). The subsequent section outlines the tasks used to evaluate executive functions and visuospatial processing.

*Working memory* was measured using a computerized visual digit span task (backwards). In this task, digits were presented sequentially in a random order in the center of the computer screen (visual angle: 0.75°) using E-Prime 3.0 (Psychology Software Tools, Inc., Pittsburgh, PA, USA). Participants had unlimited time to recall and reproduce the digit sequences in reverse order by pressing number keys on a keyboard. The sequence began with two digits and increased by one digit (up to a maximum of nine digits) each time the participant correctly reproduced the sequence twice in a row at each level. Failure to reproduce a sequence of two consecutive trials within the same level terminated the test. Digits appeared for 1,000 ms followed by an interstimulus interval of 1,000 ms with a central fixation cross. For familiarization, participants completed two practice trials with a sequence of two digits before beginning the main task. The primary outcome variable was the number of digits in the longest sequence that the participant could correctly reproduce.

*Inhibitory control* was assessed using a computerized visual-verbal Stroop color-word task (Victoria version) as described in a previous study [41]. The test comprised three conditions, each consisting of 24 stimuli. Condition I featured colored circles in green, blue, yellow, or red; condition II consisted of neutral words (loud, above, hard, or strong [in German]) colored in the same hues; and condition III involved incongruent colored words (green, blue, yellow, or red [in German]). In each condition, participants had to rapidly name the (font) color of the stimuli displayed for 2,000 ms on a computer screen to trigger the voice key. The participant’s verbal response was recorded via a microphone and manually evaluated by the test administrator. Following response evaluation, a fixation cross was presented for 1,500 ms before the next stimulus appeared. A 30-second break was provided between conditions. The Stroop task was administered using E-Prime 3.0 which controlled stimulus presentation and recorded response time (RT). RTs that were either premature (RT < 250 ms) or delayed (RT > median + 3* interquartile range) were excluded from the RT analysis as part of the data preprocessing with Excel [41]. Errors were coded as responses that were too soft for the microphone to detect, and responses that did not match the color name (e.g., “um”, incorrect color naming). To assess inhibitory control, the Stroop score was calculated using only the RTs of correct responses. It is defined as the time ratio of the color-word interference task to the color-only task (Condition III over Condition I). A smaller Stroop score indicates better performance.

*Cognitive flexibility* was assessed using the task-switching paradigm which involved two tasks and was based on the testing procedures used in previous research [53–55]. The task required participants to respond to a white digit (1–9, excluding 5) presented in the center of the screen against a black background (visual angle: 0.8°). Each digit was framed by either a solid or a dashed square (visual angle: 4.6°). If the frame was solid, participants had to judge whether the digit was greater or less than five (Task A: low/high). If the frame was dashed, they determined whether the digit was odd or even (Task B: odd/even). The task-switching paradigm was divided into six blocks. Block 1 and 2 consisted of homogeneous trials in which participants responded to only one task (pure task condition). Blocks 3 to 6 consisted of heterogeneous trials, where participants had to alternate between both tasks (mixed task condition) with switch (i.e., AB or BA) and non-switch trials (i.e., AA or BB) in accordance with the alternating runs paradigm [54, 56]. Each block contained 64 trials, resulting in 64 trials for each pure task condition (A and B) and a total of 256 trials for the mixed task condition. Participants were given a three-minute break between blocks. After a countdown in reverse order (3, 2 and 1), digits were presented for 200 ms, followed by a 2000 ms interstimulus interval from the stimulus offset of the previous trial to the subsequent onset of the next trial, with a central fixation cross displayed. Stimulus presentation and RT recording were conducted using E-Prime 3.0. Participants were instructed to place their left and right index fingers on the outer left (low/odd) and right (high/even) buttons of the Chronos box (Psychology Software Tools, Inc., Pittsburgh, PA, USA). The initial task condition in the homogeneous block (A or B) in the pre-test session was randomly assigned via randomizer.org and alternated in the post-test session. Missed responses and premature responses (RT < 100 ms) were coded as errors as part of the data preprocessing and excluded from further analysis of RT [57, 58]. RTs of correct responses were averaged for each task condition (pure A, pure B, switch trials, non-switch trials). As dependent variables, we calculated the global switch cost as the difference between the mean RT and accuracy (ACC) in heterogeneous blocks (averaged across switch and non-switch trials) compared to homogeneous blocks (averaged across pure A and pure B trials) and the local switch cost (LSC) as the difference between performance on switch trials and non-switch trials within the heterogeneous blocks. The global switch cost represents the efficiency of maintaining and selecting between multiple task sets in working memory [59, 60], while the local switch cost reflects the efficiency of activating the currently relevant task set and deactivating the task set that was relevant on the previous trial [54].

We assessed participants’ *perceptual processing* by using a visual search task, as outlined by Hommel et al. [44] and Voelcker-Rehage et al. [32]. The stimulus arrays consisted of a set of 2, 8, or 14 white-filled and unfilled circles and white-filled squares on a black background. Participants were instructed to press a right key (“M”) on the keyboard as quickly and accurately as possible when they detected a white-filled circle (target) on the screen, and to indicate that the target was absent by pressing a left key (“X”). The task included two additional conditions: In the feature search condition, the target appeared among unfilled circles (distractor I), and in the conjunction search condition, the target appeared among unfilled circles and a white-filled square (distractor II). The display area on the screen was 20 cm by 16.9 cm. Participants completed four blocks of 50 trials, which varied by set size, target presence, and search condition and were presented in a randomized order. A fixation cross was displayed for 1000 ms before each trial, followed by a random blank interval between 200 and 300 ms. The stimulus remained on the screen until a response was made or until 5000 ms had elapsed. Participants had a 30-second break between blocks. Prior to the actual test, participants completed a practice block of 20 trials to ensure full understanding of the task instruction. Stimulus presentation and RT recording were managed using Presentation^®^ (NeuroBehavioral Systems, Berkeley, CA, USA). As part of the data preprocessing, a trial was scored as an error if the response was premature (RT < 50 ms), absent (RT > 2000 ms) or wrong [32]. Perceptual processing was assessed by using RTs of correct responses and the ACC.

### Secondary measures: physical and motor performance

Participants’ muscle strength was assessed by measuring grip strength and leg extension strength. Grip strength was measured using an electronic hand dynamometer (model EH101). Participants were instructed to sit in a chair and apply maximum force when pressing the hand dynamometer. Two trials were conducted for each hand, with the maximum value used as the dependent variable.

Isometric leg extension strength (ILES) was measured using a replica of the Freiburg leg extension strength device [61]. This device works on the principle of a leg press machine and measures the extension force of the entire leg chain via two force plates, each equipped with three one-dimensional piezo force sensors (Kistler^®^, Winterthur, Switzerland). Participants lay supine on a movable padded board that was individually adjusted to ensure that both the knee and hip angles were positioned at 90 degrees. Participants were instructed to push as hard and as fast as possible with both legs against the two force plates. The force-time curves from two trials were recorded, the analogue signals were amplified and transferred to an A/D converter card, where they were digitized at a frequency of 500 Hz. The force signal was then smoothed using the Imago software package (Imago ProcessMaster, Pfister, Endingen), with a Butterworth low-pass filter (cut-off frequency 50 Hz) [61]. The maximum amplitude was used to assess maximal ILES.

Lower body functional muscle power was assessed using the five-times sit-to-stand (STS) test [62–64]. Participants were seated in a chair without armrests at a seat height of 45 cm. The stopwatch started when participants initiated the movement to stand for the first time and stopped when they sat down after completing five full stands.

Several balance tasks were administered to evaluate the diversity of balance control. The short version of the Balance Evaluation System Test (Mini-BESTest) was used to assess general balance performance [65]. The Mini-BESTest consists of 14 items (e.g., single-legged stance, Timed Up and Go test, walking over an obstacle, standing on an inclined surface) in order to evaluate postural control such as sensory orientation, anticipatory responses, and reactive postural control. Each task is scored on a 3-point ordinal scale ranging from 0 (difficult, not possible) to 2 (normal), depending on the performance of each item. The maximum possible score is 28 points [66].

In addition, normal walking speed was measured to assess physical functioning [67]. Walking speed (m/s) was recorded using the OptoGait optical detection system (Microgate Srl, Bolzano, Italy) over a distance of four meters. Participants started and ended their gait one meter before and after the measured distance to allow for acceleration and deceleration phases. Data from three trials were averaged to determine the mean usual walking speed. Participants performed all physical and motor tasks either barefoot or wearing socks.

### Exercise intervention

The exercise intervention phase took place from September to December 2023 and followed previous training protocols [41, 68] and the recommendations of Granacher et al. [69]. Training for each exercise modality was conducted in small groups of five participants, supervised by one qualified trainer. Groups commenced their training at staggered intervals following the completion of baseline assessment. The trainers recorded participants’ attendance in each session. Each intervention group completed 60-minute training sessions twice a week on non-consecutive days for a duration of 10 weeks. The intervention period was structured into a one-week introduction phase followed by three main training blocks of three weeks each. The training intensity was increased gradually from block to block. After the first, fourth, and seventh week, the individual training load for each primary exercise, such as squats, lunges or leg presses, was determined using a multiple repetition maximum test (M-RM) and adjusted accordingly. The M-RM was used to prevent injury in older and inexperienced individuals, and helped to calculate the hypothetical one-repetition maximum (1-RM) from the load and the number of repetitions according to a prediction equation for older adults [70]. Participants in the resistance training groups consistently performed the M-RM with the (unstable) equipment introduced in the initial training phase. Training adaptations were modulated by increasing the load (from 50 to 60% 1-RM) and the number of sets for the resistance training groups (MRT, T-RT). In addition, the level of instability and complexity was escalated for the MRT and BT group by varying the unstable equipment and task conditions (e.g., eyes open vs. closed). Further details can be found in Tables 1.

**Table 1:** Exercise interventions

**Warm-up/Mobility Exercises**	**Intro-phase ** **(1 week)**	**Block 1 ** **(3 weeks)**		**Block 2 ** **(3 weeks)**	**Block 3 ** **(3 weeks)**	
Warm-up	10 min (Cross-Trainer/bike/rowing ergometer)					
Mobility Exercises - 1	Hamstring-Kick (10-15x/leg), Glute Stretch (30s/side), Dead Bug (10-15x/side), Hip Mobi (10-15x/side)					
Mobility Exercises - 2	Glute Stretch (30s/side), Floor Angle (10-15x), Open Book (10-15x/side), Child to Cobra (10-15x)					
BT	4 reps per exercise with 30 s activity and 30 s rest between reps, 2 min rest between exercises					
Bipedal Stance – Exercise 1	TOGU Aero Step + wB + Eoweight shift (left/right)		SoftX coordination rocker + wB + Eo	SoftX coordination rocker + Ec	SoftX coordination rocker + Bc-t	
Bipedal Stance – Exercise 2	TOGU Aero Step + wB + Eogaze wandering to right & left, up & down		solid ground + Bc-t	Airex Balance Pad + Bc-t	Airex Balance Pad + Ec	
Single Leg Stance (2x RF, 2x LF)	solid ground + wB + Eo		Airex Balance Pad + wB + Eo	SoftX coordination rocker + wB + Eo	AirexBalance Pad + Bc-t	
Tandem Stance (in front: 2x RF, 2x LF)	solid ground + wB + Eo		Airex Balance Pad + wB + Eo	SoftX coordination rocker + wB + Eo	Airex Balance Pad + Bc-t	
MRT	2 × 12 reps (low load)		3 × 15 reps (50% 1-RM)	3-4 × 15 reps (60% 1-RM)		4 × 15 reps (60% 1-RM)
	2 min rest between sets, 5 min rest between exercises					
Squats	150° knee flex/ext angle on Airex Balance Pad	120° knee flex/ext angle on SoftX coordination rocker (round/angled)		100° knee flex/ext angle on Airex Balance Pad placed on SoftX coordination rocker (round/angled)		100° knee flex/ext angle on BOSU Ball (upside down)
Front Lunges	no additional device	Theraband Balance Pad (front foot)		TOGU Aero-Step (front foot)		TOGU Aero-Step (front foot) Airex Balance Pad (rear foot)
Core Exercise	Airex Balance Pad (under feet) (2 x 15 reps)	TOGU Dynair (under feet) Airex Balance Pad (under shoulder) (3 x 20 reps)		Crunches on BOSU Ball (4 x 20 reps)		Sit ups on BOSU Ball (4 x 20 reps)
T-RT	2 × 12 reps (low load)	3 × 15 reps (50% 1-RM)		3-4 × 15 reps (60% 1-RM)		4 × 15 reps (60% 1-RM)
	2 min rest between sets, 5 min rest between exercises					
Smith Machine	150° knee flex/ext angle	120° knee flex/ext angle		100° knee flex/ext angle		
Single Leg Press	90° knee flex/ext angle					
Core Exercise	Bridge exercise (2 × 15 reps)	Bridge exercise (3 × 20 reps)		Crunches (feet on the ground) (4 × 20 reps)		Sit ups (feet on the ground) (4 × 20 reps)

1-RM One Repetition Maximum, BT Balance Training, Bc-t Ball catching and throwing, Eo Eyes open, Ec Eyes closed, LF Left Foot, M-RM Multiple Repetition Maximum, MRT MetastableResistance Training, RF Right Foot, T-RT Traditional Resistance Training, wB without Ball

After week one, four and seven the training load was increased following M-RM testing and 1-RM prediction using the equation provided by Tan et al. (2015) for each main exercise. The M-RM test was conducted in the intro-phase condition for every group

#### MRT

Participants performed free weight squats on an instability device (e.g., foam pad, BOSU ball) and front lunges with instability devices under one or both feet. Secondary exercises included core exercises (bridge exercise, crunches) using instability devices.

#### T-RT

This group performed squats on the Smith machine with the barbell positioned on the hips and single leg exercises on a leg press. Secondary exercises focused on core strengthening (bridge exercise, crunches).

#### BT

The balance group performed four exercises per session in three different stances (bipedal, tandem, single leg) using different instability devices and additional tasks (e.g., eyes open vs. closed, catching and throwing a ball).

Regardless of the training modality, all groups began each session with a 10-minute warm-up on a cycle ergometer or cross trainer. Exercises to mobilize the target muscle groups were incorporated between the warm-up and the main exercises, as well as at the end of the session. Compliance calculated as the percentage of total training sessions attended, was derived from the attendance records.

### Data analysis

The statistical analysis aimed to evaluate whether the metabolic and coordinative demands of MRT provide beneficial effects on cognitive performance compared to traditional balance and resistance training. To achieve this, we used linear mixed-effects models. Separate models were developed to evaluate the cognitive functions of interest (inhibitory control, working memory, cognitive flexibility, perceptual processing). Each model included time (pre, post) and group (BT, T-RT, MRT as reference) as fixed effects, along with their interaction terms. Additionally, we considered age (continuous), gender (categorical; 1 = male, 2 = female), MoCA score (continuous), baseline physical activity level (continuous) and training attendance (continuous) as covariates, also treated as fixed effects. Age and gender were included due to their roles as moderators in training effects, while MoCA score, activity level and attendance were added to account for possible differences among training groups. Non-significant covariates (i.e., *p* > 0.05) were removed from the final models individually through a backwards exclusion procedure. Models were developed separately for RT and ACC.

For the cognitive tasks, statistical analysis was performed with the data from 83 participants, with the exception of the visual search task where data were available from 82 individuals owing to one excluded individual exceeding a 50% error rate. In addition, data from only 74 participants were available for the analysis of global and local switch costs of the task-switching paradigm. Nine subjects were excluded owing to incomplete testing procedures or failure to achieve 50% ACC in either the averaged homogeneous or heterogeneous block of the pre- or post-test sessions. For the physical and motor tasks, data from 81 participants were analyzed as two individuals were unable to attend the post-intervention session owing to illness or scheduling conflicts (see Fig. 1).

The visual search task analysis also focused on RT and ACC for the 14-stimuli display size condition. Previous research showed a significant association between physical exercise and the performance in task conditions that require greater interference control [32, 59]. The analyzed 14-stimuli trials included conjunction and feature search conditions in order to include a sufficient number of trials per person in the analysis, with trial numbers ranging from 38 to 84 (pre-test) and 42 to 82 (post-test).

To investigate the effects of the different training modalities on physical and motor performance, linear mixed-effects models were applied using the same fixed effects outlined above for cognitive performance. The time to complete the Timed Up and Go test, conducted as a component of the Mini-BESTest, was separately analyzed as a measure of proactive balance [64, 71].

One-way ANOVAs were performed to compare group characteristics at baseline [72]. Significant differences between groups were further analyzed using a post-hoc Tukey’s HSD (honestly significant difference) test. Linear mixed-effects models were computed using Rstudio (Posit Software, PBC, version 2025.05.1) and the lmerTest package [73]. The alpha level was set to 5%. Standardized beta coefficients (*β*) and the corresponding 95% confidence intervall (CI) are provided. Graphical results were plotted using a script from Loffing [74].

## Results

A total of 83 participants successfully completed the training phase with an overall participation rate of more than 90% across all training groups. Group characteristics are detailed in Table 2. The results from the one-way ANOVA revealed a significant difference in the MoCA scores between groups, *F*(2,80) = 3.39, *p* = 0.039, $\:{\eta\:}_{p}^{2}$ = 0.08. Subsequent analysis using Tukey’s HSD post hoc test showed that, on average, participants in the MRT group had significantly lower MoCA scores compared to those in the T-RT group (*p* = 0.031). There were no significant differences in the baseline activity level, *F*(2,80) = 1.59, *p* = 0.211, $\:{\eta\:}_{p}^{2}$ = 0.04, and the training attendance, *F*(2,80) = 0.48, *p* = 0.623, $\:{\eta\:}_{p}^{2}$ = 0.01, between groups.

**Table 2:** Characteristics of participants at baseline

Characteristics	BT (*n* = 28)		MRT (*n* = 29)		T-RT (*n* = 26)	
	*M*	*SD*	*M*	*SD*		*SD*
Female (*n*)	18		20		17	
Age (years)	70.6	4.7	70.5	4.8	70.4	4.2
Height (cm)	168.5	8.8	168.7	10.9	168.0	8.8
Weight (kg)	72.3	16.4	70.8	15.6	70.6	11.5
MoCA score	26.9	2.2	26.3	1.8	27.7	1.8
Activity level (MET-h/week)	83.0	50.1	68.0	47.9	61.6	37.0
Attendance (%)	93.0	7.1	91.7	7.7	91.0	8.9

*BT* Balance Training, *MET* Metabolic Equivalent of Tasks, *MoCA* Montreal Cognitive Assessment, *MRT* Metastable Resistance Training, *T-RT* Traditional Resistance Training

### Primary outcome measures: cognitive and executive performance

**Fig. 2:**
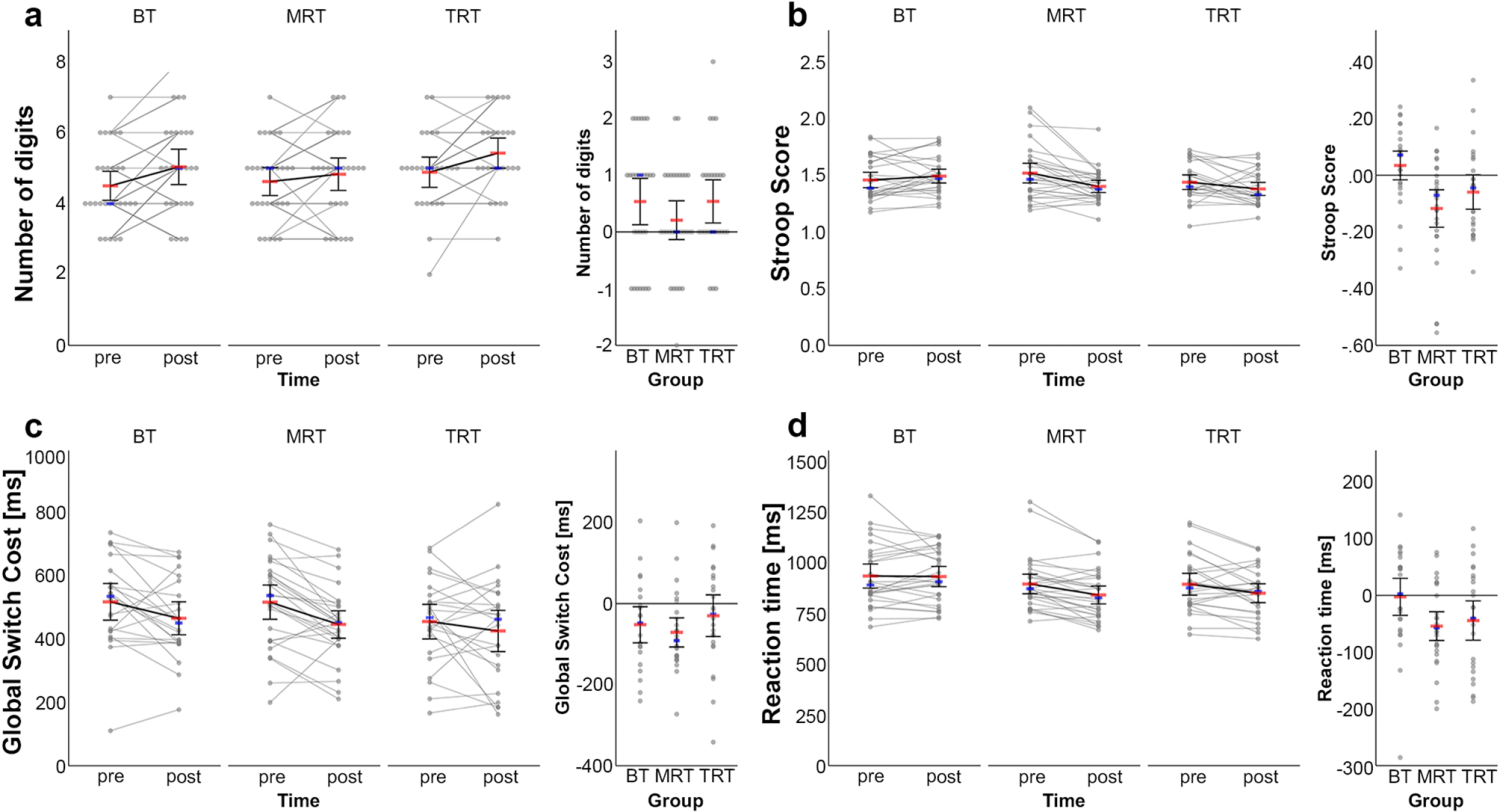
Line Plots of Task Performance in the **a**) Digit Span Task-Backwards, **b**) Stroop Task, **c**) Task-Switching Paradigm and **d**) Visual Search Task from Pre-intervention and Post-intervention Measurements Note. BT = balance training; MRT = metastable resistance training; T-RT = traditional resistance training

Mean values and standard deviations of primary outcome parameters are presented in Table 3. Figure 2 shows the change in performance in the cognitive tasks from pre- to post-intervention measurement.

**Table 3:** Means and standard deviations of cognitive parameters

Variable	BT					MRT					T-RT				
	*n*	pre		post		*n*	pre		post		*n*	pre		post	
		*M*	*SD*	*M*	*SD*		*M*	*SD*	*M*	*SD*		*M*	*SD*	*M*	*SD*
DSB															
Sequence length	28	4.50	1.11	5.04	1.35	29	4.62	1.08	4.83	1.26	26	4.88	1.11	5.42	1.10
Stroop															
RT (ms)	28					29					26				
Circle		659.87	71.45	622.68	63.59		656.51	105.59	664.39	106.08		667.04	134.94	672.65	90.14
Neutral		822.57	104.48	781.63	100.83		810.34	115.71	783.42	121.26		803.72	130.05	790.31	127.41
Incongruent		963.29	119.36	931.38	107.71		988.42	162.23	929.98	159.68		960.23	153.03	928.21	156.97
Stroop score		1.46	0.18	1.49	0.16		1.52	0.24	1.40	0.15		1.44	0.17	1.38	0.15
TSP															
RT (ms)	23					27					24				
Homogeneous		581.08	110.93	595.81	101.91		592.34	80.22	607.96	85.16		625.80	69.70	634.85	103.28
Heterogeneous		1101.03	193.79	1063.70	180.43		1111.35	165.98	1056.13	136.40		1083.04	133.12	1062.31	174.41
Switch		1156.01	218.48	1114.78	206.93		1167.72	190.11	1113.81	148.43		1133.71	123.67	1098.66	166.50
Non-switch		1046.05	175.61	1012.62	163.67		1054.99	150.78	998.45	137.16		1032.38	156.33	1025.96	191.68
GSC		519.95	142.97	467.89	128.17		519.01	144.80	448.17	115.49		457.24	137.15	427.46	164.11
LSC		109.96	83.19	102.16	94.88		112.73	86.95	115.36	85.25		101.33	92.60	72.70	85.16
ACC (%)	23					27					24				
Homogeneous		0.95	0.05	0.97	0.03		0.95	0.04	0.98	0.03		0.97	0.02	0.98	0.02
Heterogeneous		0.80	0.11	0.87	0.09		0.81	0.12	0.86	0.10		0.81	0.13	0.86	0.13
Switch		0.79	0.12	0.86	0.10		0.79	0.13	0.85	0.11		0.80	0.13	0.86	0.13
Non-switch		0.81	0.11	0.88	0.09		0.83	0.12	0.87	0.10		0.82	0.13	0.86	0.12
GSC		−0.15	0.12	−0.11	0.08		−0.14	0.11	−0.11	0.09		−0.16	0.13	−0.12	0.13
LSC		−0.01	0.05	−0.02	0.05		−0.04	0.05	−0.02	0.03		−0.02	0.04	−0.002	0.04
VST															
RT (ms)	27					29					26				
All trials		935.79	157.23	933.33	132.49		896.38	132.52	842.31	121.24		895.18	140.07	850.92	120.87
14 stimuli		1161.07	208.00	1138.10	175.18		1061.40	158.30	1014.49	162.66		1050.77	161.04	1009.80	149.64
ACC (%)	27					29					26				
All trials		97.15	2.76	96.89	3.57		97.14	2.85	98.45	1.55		97.38	3.69	98.58	3.10
14 stimuli		93.12	7.37	92.94	8.36		95.19	6.58	97.21	3.92		95.07	7.59	96.88	6.99

*ACC* accuracy, *BT* balance training, *DSB* digit span backwards, *GSC* global switch cost, *MRT* metastable resistance training, *RT* reaction time, *T-RT* traditional resistance training, *TSP* task-switching paradigm, *VST* visual search task

#### Working memory

Using MRT as the reference group, linear mixed-effects model analysis did not reveal a significant main effect of time, *t*(80) = 1.10, *p* = 0.275, *β* = 0.09, 95% CI [−0.16, 0.58], nor an interaction effect between MRT and BT, *t*(80) = 1.22, *p* = 0.224, *β* = 0.14, 95% CI [−0.20, 0.86], or between MRT and T-RT, *t*(80) = 1.21, *p* = 0.229, *β* = 0.14, 95% CI [−0.21, 0.87], on the sequence length of the digit span backwards task. Covariates including age, gender, MoCA score, activity level, and attendance were non-significant (*p* > 0.05) and therefore removed. Estimates of the linear mixed-effects models are presented in Table 4.

**Table 4:** Estimates of fixed effects on cognitive performance with MRT as reference group

			TSP				VST			
	Sequence length	Score	RT		ACC		RT		ACC	
			GSC	LSC	GSC	LSC	All trials		All trials	
Intercept	4.41***	2.25***	589.85***	110.11***	−0.10	−0.06***	−8.81	59.51	107.61***	122.12***
Time	0.21	−0.12***	−70.84***	2.62	0.02	0.02	−54.07***	−46.92*	1.31**	2.02*
Group [BT]	−0.45	−0.20**	−17.83	7.65	−0.06	0.05*	−15.24	72.40	1.62	0.21
Group [T-RT]	−0.07	−0.11	−102.84	19.85	−0.07	0.02	−9.22	−14.60	0.34	0.03
Time x Group [BT]	0.33	0.15***	18.77	−10.42	0.02	−0.03	51.61*	23.94	−1.57**	−2.19
Time x Group [T-RT]	0.33	0.06	41.06	−31.25	0.01	−0.00	9.82	5.94	−0.12	−0.21
Age (years)					−0.01*		13.60***	14.87***	−0.17*	−0.41**
Gender					−0.05*					
MoCA score		−0.02*			0.02**					
Activity level (MET-h/week)										
Attendance (%)										

* ACC* accuracy, *BT* balance training, *DSB* digit span backwards, *GSC* global switch cost, *LSC* local switch cost, *MET* Metabolic Equivalent of Tasks, *MoCA* Montreal Cognitive Assessment, *MRT* metastable resistance training, *RT* reaction time, *T-RT* traditional resistance training, *TSP* task-switching paradigm, *VST* visual search task

*p ≤ 0.05, **p ≤ 0.01, ***p ≤ 0.001

#### Inhibitory control

A significant main effect of time indicated that the Stroop score decreased over time, *t*(80) = −3.92, *p* < 0.001, *β* = −0.32, 95% CI [−0.49, −0.16], reflecting improved inhibitory control. The time-by-group interaction was significant when comparing MRT with BT, *t*(80) = 3.56, *p* < 0.001, *β* = 0.42, 95% CI [0.19, 0.65], indicating significantly greater improvements in inhibitory control following MRT. In contrast, the interaction did not reach significance when comparing MRT and T-RT, *t*(80) = 1.30, *p* = 0.198, *β* = 0.16, 95% CI [−0.03, 0.14]. MoCA score showed a significant effect, *t*(79) = −2.60, *p* = 0.011, *β* = −0.25, 95% CI [−0.44, −0.06], suggesting that participants with higher MoCA scores exhibited lower Stroop scores, implying better inhibitory control.

#### Cognitive flexibility

The linear mixed-effects model revealed a significant main effect of time on the GSC RT in the task-switching paradigm, *t*(71) = −3.32, *p* = 0.001, *β* = −0.25, 95% CI [−0.40, −0.10], but no significant interaction between groups and time (*p* > 0.19). Pre-to-post-test changes in GSC ACC were not significant, *t*(71) = 0.97, *p* = 0.338, *β* = 0.09, 95% CI [−0.10, 0.29]. However, covariates including age, gender, and MoCA score were significant, indicating smaller switch cost in accuracy between heterogeneous and homogeneous blocks for younger male participants with higher MoCA scores. LSC RT did not demonstrate a significant change over time, *t*(71) = 0.16, *p* = 0.871, *β* = 0.02, 95% CI [−0.17, 0.20], nor a significant time-by-group interaction (*p* > 0.19). A significant main effect of group showed that LSC ACC differed between MRT and BT, *t*(103) = 2.04, *p* = 0.044, *β* = 0.24, 95% CI [0.00, 0.10], with participants in the BT group showing smaller switch cost between switch trials and non-switch trials.

#### Perceptual processing

A significant main effect of time was found on response behavior in all trials, indicating a decrease in RT, *t*(79) = −3.54, *p* = 0.001, *β* = −0.20, 95% CI [−0.31, −0.09], and an increase in ACC over time, *t*(79) = 3.24, *p* = 0.002, *β* = 0.22, 95% CI [0.09, 0.35]. A significant time-by-group interaction was observed when comparing MRT and BT for both RT, *t*(79) = 2.35, *p* = 0.020, *β* = 0.19, 95% CI [0.03, 0.35], and ACC, *t*(79) = −2.69, *p* = 0.009, *β* = −0.26, 95% CI [−0.45, −0.07], suggesting larger improvements in response behavior for MRT. Response behavior in trials with 14 stimuli improved significantly from pre- to post-test, for RT, *t*(79) = −2.05, *p* = 0.044, *β* = −0.13, 95% CI [−0.26, 0.00], and for ACC, *t*(79) = 2.42, *p* = 0.018, *β* = 0.14, 95% CI [0.03, 0.26]. Time-by-group interactions did not reach significance. Moreover, age significantly influenced RT, *t*(78) = 4.91, *p* < 0.001, *β* = 0.45, 95% CI [0.27, 0.63], and ACC, *t*(78) = −2.53, *p* = 0.013, *β* = −0.25, 95% CI [−0.45, −0.05], in all trials as well as in trials with 14 stimuli (RT: *t*(78) = 4.20, *p* < 0.001, *β* = 0.38, 95% CI [0.20, 0.56]; ACC: *t*(78) = −2.65, *p* = 0.01, *β* = −0.26, 95% CI [−0.46, −0.07]). These effects indicate slower response times and lower response accuracy with increasing age.

### Secondary outcome measures: physical and motor performance

Mean values and standard deviations of physical outcome parameters are listed in Table 5. Estimates of the linear mixed-effects models are presented in Table 6. Since no significant interaction effects were found, only the significant main effects of time and significant covariates are described below.

**Table 5:** Means and standard deviations of physical and motor parameters

Variable	BT (*n* = 27)					MRT (*n* = 29)					T-RT (*n* = 25)				
	pre		post		Δ %	pre		post		Δ %	pre		post		Δ %
	*M*	*SD*	*M*	*SD*		*M*	*SD*	*M*	*SD*		*M*	*SD*	*M*	*SD*	
Strength															
Grip strength															
Right hand (kg)	31.88	10.80	32.28	10.01	2.48	29.28	10.39	30.57	10.15	5.28	30.95	7.81	31.71	8.23	2.48
Left hand (kg)	30.51	11.34	31.64	11.86	3.74	29.09	10.54	29.52	10.34	2.46	28.70	8.88	29.75	8.98	4.87
ILES															
Right leg (kg)	40.24	15.72	50.68	18.47	31.19	39.45	13.18	50.50	14.33	32.21	39.73	11.11	48.91	14.46	26.83
Left leg (kg)	39.03	16.26	47.72	17.40	29.03	35.74	13.48	46.00	13.21	37.14	34.65	10.45	43.40	11.04	29.53
Sit-to-stand test (s)	10.46	2.65	10.39	3.05	−0.18	11.07	2.65	10.64	2.15	−2.12	10.17	2.11	10.40	2.84	1.61
Balance															
Mini-BESTest (points)	23.63	1.62	24.70	1.77	4.78	23.45	2.11	24.38	1.82	4.51	23.68	2.14	24.76	1.79	5.07
TUG (s)	8.33	1.10	7.91	1.09	−4.81	7.99	0.93	7.30	0.92	−8.28	8.03	1.12	7.67	0.94	−4.01
Usual walking speed (m/s)	1.22	0.19	1.31	0.18	8.14	1.34	0.19	1.45	0.20	8.65	1.29	0.18	1.35	0.15	6.26

*BT* balance training, *ILES* isometric leg extension strength, *Mini-BESTest* short version of the Balance Evaluation System Test, *MRT* metastable resistance training, *T-RT* traditional resistance training, *TUG* timed up and go test

**Table 6:** Estimates of training group and participant characteristics on physical and motor performance with MRT as reference group

	Strength					Balance		
	Grip strength		ILES		Sit-to-stand test	Mini-BESTest	TUG	Walking speed
	right	left	right	left				
Intercept	59.92***	59.83***	57.14*	29.63*	0.47	32.29	3.29*	0.61*
Time	1.29**	0.43	11.05***	10.27***	−0.43	0.93*	−0.68***	0.11***
Group [BT]	2.91	−0.08	−0.16	4.05	−1.05	−0.10	0.02	−0.10
Group [T-RT]	2.13	−1.06	−0.36	−0.97	−1.57	0.13	−0.33	−0.04
Time x Group [BT]	−0.90	0.65	−0.74	−1.82	0.38	0.20	0.28	−0.02
Time x Group [T-RT]	−0.56	0.60	−1.91	−1.61	0.66	0.13	0.33	−0.04
Age			−0.55*		0.16**	−0.14***	0.08***	
Gender	−17.85***	−18.45***	−20.78***	−20.33***				
MoCA score			1.72**	1.15*				0.02*
Activity level (MET-h/week)	−0.03*							
Attendance (%)								

*BT* balance training, *MET*Metabolic Equivalent of Tasks, *Mini-BESTest* short version of the Balance Evaluation System Test, *MoCA *Montreal Cognitive Assessment, *MRT* metastable resistance training, *T-RT* traditional resistance training, *TUG* timed up and go test

*p ≤ 0.05*, p *≤ 0.01, ***p ≤ 0.001

#### Grip strength

Linear mixed-effects model analysis revealed a significant main effect of time for the right hand, *t*(78) = 3.03, *p* = 0.003, *β* = 0.07, 95% CI [0.02, 0.11], but no significant improvement in grip strength for the left hand, *t*(78) = 0.74, *p* = 0.465, *β* = 0.02, 95% CI [−0.04, 0.08]. Covariates including gender, and physical activity level were significant, indicating lower hand grip strength among female (right: *t*(78) = −15.09, *p* < 0.001, *β* = −0.88, 95% CI [−0.99, −0.76]; left: *t*(79) = −15.05, *p* < 0.001, *β* = −0.85, 95% CI [−0.96, −0.74]), and more active participants (only right hand: *t*(78) = −2.08, *p* = 0.04, *β* = −0.12, 95% CI [−0.24, −0.01]).

#### Isometric leg extension strength (ILES)

ILES demonstrated a significant increase following MRT in the right leg, *t*(78) = 6.77, *p* < 0.001, *β* = 0.36, 95% CI [0.26, 0.47], and in the left leg, *t*(77) = 6.71, *p* < 0.001, *β* = 0.35, 95% CI [0.25, 0.46]. Covariates including gender (right: *t*(77) = −8.89, *p* < 0.001, *β* = −0.64, 95% CI [−0.78, −0.50]; left: *t*(77) = −9.24, *p* < 0.001, *β* = −0.66, 95% CI [−0.80, −0.52]), age (only right leg: *t*(77) = −2.27, *p* = 0.026, *β* = −0.16, 95% CI [−0.31, −0.02]), and MoCA were significant (right: *t*(77) = 2.96, *p* = 0.004, *β* = 0.22, 95% CI [0.07, −0.37]; left: *t*(77) = 2.09, *p* = 0.040, *β* = 0.16, 95% CI [0.01, −0.30]).

#### Lower body functional muscle power

The sit-to-stand time did not significantly improve from pre- to post-test following MRT, *t*(78) = −1.31, *p* = 0.193, *β* = −0.08, 95% CI [−0.21, 0.04], but was significantly influenced by age, *t*(79) = 2.74, *p* = 0.008, *β* = 0.28, 95% CI [0.08, 0.47]. The positive coefficient indicates slower times with increasing age.

#### Balance

The total score of the Mini-BESTest significantly increased by roughly one point from pre- to post-testing following MRT, *t*(78) = 2.56, *p* = 0.013, *β* = 0.24, 95% CI [0.05, 0.43]. Age showed a significant impact on performance in the Mini-BESTest, *t*(78) = −3.79, *p* < 0.001, *β* = −0.32, 95% CI [−0.49, −0.15], suggesting lower scores with increasing age.

#### Proactive balance

Significant reductions were observed in mean Timed Up and Go time following MRT, *t*(78) = −5.42, *p* < 0.001, *β* = −0.33, 95% CI [−0.44, −0.21]. Additionally, proactive balance was significantly influenced by age, *t*(79) = 3.45, *p* < 0.001, *β* = 0.33, 95% CI [0.14, 0.52], demontrating longer times to complete the Timed Up and Go test with increasing age.

#### Physical functioning

Linear mixed-effects model analysis revealed a significant main effect of time, *t*(78) = 4.61, *p* < 0.001, *β* = 0.28, 95% CI [0.16, 0.40]. Faster usual walking speed was associated with higher MoCA scores, *t*(78) = 2.45, *p* = 0.016, *β* = 0.25, 95% CI [0.05, 0.44].

## Discussion

The main objective of this study was to investigate the effect of MRT with its combined metabolic and coordinative demands, particularly on cognitive performance in older adults. The results suggest that MRT led to greater improvements in inhibitory control and perceptual processing speed compared to traditional balance training (BT). These findings support the notion that the combined metabolic and cognitive demands of MRT can improve performance on cognitive tasks requiring perceptual processing, executive control, and attention. However, improvements on the executive task related to inhibitory control were observed only in comparison to balance training and not resistance training, indicating that MRT impacts cognition in a task-specific way. In the following sections, the effects of MRT on cognitive performance are discussed in more detail.

### Effects of metastable resistance training on cognition

Our results partially confirm those reported in previous studies. Similar to the observation made by Eckardt et al. [41], older adults showed improved inhibitory control following MRT. We used the exactly same Stroop task as Eckardt et al. [41], allowing a direct comparison of results. However, it is worth noting that the performance levels, including reaction times (RT) and Stroop scores, were slightly lower in our study than those reported previously [41] (RT_circle_ = 623.3 ms; RT_neutral_ = 754.9 ms; RT_incongruent_ = 903.2 ms; Stroop score: pre, 1.45; post, 1.35). Contrary to the findings of Eckardt et al. [41], the present study did not show a more beneficial effect of MRT on inhibitory control compared to T-RT. However, incorporating an additional active control group revealed that MRT resulted in better performance in the Stroop task compared to BT.

In line with the methodology outlined before [41], our study used a basic cognitive task to assess perceptual processing speed along with other core executive functions. Participants who engaged in MRT showed enhanced response behavior, characterized by decreased response times and simultaneously increased accuracy across all trials of the visual search task, compared to participants of the BT group.

In contrast to Eckardt et al. [41] and conceptual models [45], MRT did not show a significant effect on performance in a working memory task involving information manipulation. Training types with coordinative demands appear to have specific effects on working memory by stimulating the brain and promoting cognitive processing, particularly the use of shared neural substrates for cognitive and motor tasks facilitating the allocation of attentional resources [45]. The lack of a significant effect may be due to the different digit span task variant used: we employed a computerized visual digit span task (backwards) instead of the previously used auditory verbal Digit Memory Task including forward and backward span [41]. These two digit span tasks not only vary in the input modality and response code but may also require different stimulus processing [75]. In addition, external factors such as participants’ manual motor abilities and technological skills may influence outcomes when manual responses are required with a computer keyboard [76]. The differences in modality (visual-manual vs. auditory-verbal), the outcome measure (sequence length vs. standard score) and participants’ technological skills may contribute to different results on the effect of MRT on working memory performance. Nonetheless, the general impact of exercise on working memory remains unclear in current research. For instance, Northey et al. [77] revealed pronounced effects of resistance exercise on working memory, while Landrigan et al. [78] found no statistically significant effect of resistance training on working memory performance in their meta-analysis. However, they revealed that the type of working memory task (verbal vs. visuospatial) acts as a moderator, with slightly more improvements noted in visuospatial working memory tasks [78]. To investigate whether effects of MRT are task-specific, particularly concerning further types of working memory tasks, future studies could utilize memory tasks characterized by verbal fluency, language processing, or visuospatial processing.

In terms of cognitive flexibility, the lack of significant effects did not provide an advantage of MRT over other training programs on global and local switch costs. It is worth noting that a similar lack of group differences was reported by Eckardt et al. [41], who assessed cognitive flexibility using a paper-and-pencil trail-making task.

The absence of significant effects on working memory and cognitive flexibility could stem from limited statistical power due to the small sample size, which was likely only adequate to detect medium-to-large effects (*d* = 0.7). A meta-analysis showed that resistance training positively influenced cognitive outcomes, with an overall effect size of SMD = 0.71; however, most individual studies in the analysis reported smaller effects [78].

All in all, our findings indicate that MRT can improve performance particularly in cognitive tasks requiring perceptual and executive processing, such as attention. This may be related to a direct effect on cognition due to the cognitive demands inherent in metastable resistance exercise [39]. However, it is surprising that the effects of MRT were only significant compared to BT, as balance and coordinative exercises in particular are considered cognitively demanding [18, 23] and showed trends of superior effectiveness on cognitive functions in healthy individuals [79]. According to Netz [23] and Tomporowski and Pesce [24], intensity and complexity appear to be the key mechanisms driving training effects. Our results suggest that the intensity component predominantly drives improvements in inhibitory control and perceptual processing, with additional improvements through complexity introduced by balance requirements. Surprisingly, task complexity alone, as imposed by BT, was not sufficient to improve inhibitory control or perceptual processing speed. However, traditional BT, which essentially involves static balance tasks [80], incorporates less task complexity compared to MRT. In MRT, the dynamic movements on unstable surfaces complicate the coordination by acting forces [37] and appear to require larger cognitive control compared to static balance tasks [81].

Furthermore, our results suggest that balance training was not sufficient to improve certain cognitive functions over a 10-week period. This finding may be attributable to the low intensity of the postural tasks, as also argued by Iuliano et al. [82]. While balance training in general can be cognitively demanding – as highlighted in previous studies – its cognitive effects may depend strongly on task characteristics and skill that are targeted. A meta-analysis indicated that moderate- or high-intensity exercise is more beneficial for cognitive outcomes than low-intensity exercise [77]. However, the moderating effect of intensity on cognitive improvements has not been consistently observed [33, 79, 83]. In contrast, coordination training has been reported to improve perceptual speed after a 12-month intervention [32]. The differences between our and their study lie not only in the duration of the training period but also in the content of the training. Our study focused only on balance exercises, whereas Voelcker-Rehage et al. [32] implemented a more diverse regimen, including exercises for eye-hand coordination, leg-arm coordination, spatial orientation, and reaction to people and moving objects, in addition to balance exercises.

To date, further comparative studies investigating the effects of MRT on cognitive performance in older, healthy adults remain still limited. For example, Mehranian et al. [84] found that older individuals showed improved short-term memory after 4 weeks of MRT with and without blood flow restriction. However, in a study by Cavalcante et al. [42], older participants with cognitive complaints did not show significant improvements in global cognition or specific cognitive subdomains after participating in either T-RT or MRT compared to a control group.

The precise mechanisms underlying the beneficial effects of MRT on response inhibition remain largely unexplored. To date, no comprehensive studies have analyzed the cortical adaptations in older adults following long-term MRT. However, findings from studies with young adults engaging in acute metastable resistance exercises and postural control tasks may provide clues. For example, increased pupil size observed during acute resistance exercise on unstable devices suggests increased activity in several neuromodulatory systems [40], such as the activation of the locus coeruleus and the release of the neurotransmitter norepinephrine [85]. Interestingly, changes in pupil size have been reported to predict Stroop task performance [86]. In addition, data from electroencephalography and neuroimaging studies have demonstrated that postural balance tasks not only increased activity in the occipital and parietal cortices [87] but also in frontal brain regions [88–91]. These regions, which include the dorsolateral prefrontal cortex and the anterior cingulate cortex, are critical for response inhibition [92, 93]. Furthermore, the link between performance in balance control and inhibition highlights the cognitive benefits potentially associated with engaging in MRT [94].

In conclusion, our study contributes new insights into the effects of coordinative and metabolic demands within physical exercise, suggesting that this combination may lead to greater improvements in response inhibition and perceptual processing speed when contrasted with BT, charaterized by coordinative but low metabolic demands. Although coordinative demands are typically associated with MRT and BT, T-RT may also involve motor learning for some participants in our study, as it introduced a new form of exercise that requires cognitive control [95] and the mastery of proper technique to prevent potential pain or injury.

### Effects on physical and motor performance - secondary outcome measures

As secondary outcomes, we examined physical and motor performance parameters that are integral components of the National Geriatric Assessment [96]. The results indicate that all training programs were effective in improving physical and motor performance, including muscle strength as well as proactive balance and walking speed.

We found no interaction effects in the strength and balance parameters assessed, despite differences in the strength and balance requirements of the training programs. However, this finding is in line with expectations, as T-RT is known to positively influence strength parameters and also improve both static and dynamic postural control [97]. Similarly, BT not only improves balance parameters but has also been shown to increase maximal strength in older adults [61].

In our study, the BT group showed high improvements in ILES, with a percent change of 31% for the right leg and 29% for the left leg from pre- to post-test. Calculating the standardized difference in means (*SDM*; the mean difference divided by the pre-test standard deviation [98]) indicates small to moderate effects of BT on leg strength (*SDM*_R_ = 0.68, *SDM*_L_ = 0.54). These effects are smaller than the very large effect observed in a previous study (*SDM* = 2.95) [61]. Meanwhile, the T-RT resulted in ILES gains of 28% for the right leg and 30% for the left leg, corresponding to moderate effects (*SDM*_R_ = 0.81, *SDM*_L_ = 0.80). These effects are smaller than the extremely large effect demontrated by Granacher et al. [97] in older men (*SDM* = 5.54). However, the reference studies examined ILES bilaterally [61, 97]. Remarkably, the MRT group achieved improvements of 32% (*SDM* = 0.84) for the right leg and 37% (*SDM* = 0.76) for the left leg. These improvements are higher than the 15% (*SDM* = 0.35) reported by Eckardt [68], who notably used a different testing method to assess ILES. Overall, the observed increases in maximal strength can be attributed to both muscular and neural adaptations, including improved intra- and intermuscular coordination, as documented in the literature [99–102]. While BT has been reported to significantly improve intermuscular coordination in older adults [61], it has been suggested that MRT imposes a larger load due to a higher degree of metastability, thus providing better opportunities for training adaptations in the neuromuscular and balance systems [34].

Although not an explicit objective of the training program, hand grip strength increased by 2.5 to 5.3% in all groups, with significant gains observed in the right hand. While Eckardt [68] reported no significant increases in hand grip strength after 10 weeks of resistance training with varying degrees of metastability, Pirauá et al. [103] found improvements in hand grip strength for both the right and left hand after 24 weeks of whole-body strength training including upper limp exercises (e.g., unilateral row with dumbbells) on both stable and unstable surfaces. Unlike other studies reporting significant improvements in sit-to-stand performance after T-RT and MRT [68, 104, 105], our study did not show significant improvements over time for any group.

Overall balance performance improved significantly in all groups, with an average increase of one point in the Mini-BESTest scores. However, in this respect, comparative studies investigating the impact of exercise on the Mini-BESTest performance in healthy community-dwelling older adults are limited. For instance, older nursing home residents showed an improvement of five points after eight weeks of the Otago exercise program [106]. However, these participants started from a much lower baseline score of 14.6 points compared to 23.8 points in our study. Importantly, a change in performance by one point must be considered negligible and may be attributed to measurement error because it is below the smallest detectable change of 3.3 points reported for older people with an increased fall risk [107]. Previous studies on MRT utilized different clinical test batteries to assess overall balance performance, such as the Berg Balance Scale, and reported significantly improved balance performance after an intervention period of 24 weeks [108].

Aditionally, proactive balance and physical functioning were assessed using Timed Up and Go test and usual walking speed. The time to complete the Timed Up and Go test was reduced by 4.0 to 8.3% in the post-test session without a significant interaction effect. These findings are consistent with previous MRT studies [42, 68, 105, 109]. For example, Eckardt [68] observed similar improvements of 6 to 8% after 10 weeks of resistance exercise with varying degrees of metastability. Conversely, Eckardt and Rosenblatt [104] reported no significant time effects for T-RT and MRT on Timed Up and Go performance. Javadpour et al. [110] found a 6.5% reduction in Timed Up and Go completion time after 6 weeks of single-task BT involving static and dynamic walking tasks.

In terms of physical functioning, improvements in normal walking speed ranged from 6 to 9% across training modalities and were slightly higher than the average increase of 5.8% or 0.07 m/s observed after resistance, coordination, and multimodal training in older adults [111]. Nevertheless, percentage improvements were still smaller compared to other studies that included dynamic walking tasks in the BT program [110].

To sum up, all three training programs had a positive effect on the physical and motor performance of the older participants included in this study. With the exception of the sit-to-stand test, significant improvements were observed from pre- to post-test session in the strength and balance tests. However, no interaction effects were found, probably because the test tasks were not part of the training programs. Transfer effects of strength and balance exercises to non-trained test tasks are known to be limited [91].

### Limitations

Due to the inclusion and exclusion criteria, the study participants had a good physical baseline level. None of the participants had a baseline walking speed below 0.8 m/s, which is considered an indicator of sarcopenia [112] or reduced mobility [113]. Therefore, our results may not be generalized to frail older adults with health issues or to patients with neurological diseases, and there may be a selection bias towards active and cognitively healthy older adults. Future research could consider including individuals with Parkinson’s disease or mild cognitive impairment to gain a more comprehensive understanding of the beneficial effects of MRT on cognition, as other studies have already demonstrated that MRT is applicable to different groups of individuals [42, 114–116].

All cognitive and executive function tasks were computerized. Computerized tasks ensure high standardization as well as objective and efficient data collection. In the task-switching paradigm, we were able to reduce the number of keys significantly compared to a keyboard by using the Chronos Box with only five keys. In the visual search task, the required keys were color-coded to facilitate operation. However, limited familiarity with computer-based tasks may also have contributed to the need to exclude some participants’ data from the analysis, further reducing the number of data sets available for analysis.

The lack of interaction effects between training programs may also be attributed to the relatively short intervention duration of 10 weeks. As concluded in reviews examining the long-term effects of resistance training on cognitive function, the duration of the intervention is a critical variable for the outcomes tested [26, 117]. Interventions lasting less than six months often produce inconsistent results regarding their effects on cognitive function. For example, studies lasting longer than 16 weeks have shown positive effects on executive functions, whereas those lasting less than 12 weeks have failed to show any significant effects [117]. Therefore, in addition to exercise intensity and task complexity, the duration of the intervention may play a critical role in the interaction between exercise and cognition. Due to the absence of a passive control group, repetition effects from familiarization with the test environment in cognitive and motor tasks cannot be entirely ruled out. However, active control groups account for training-related side effects, such as adherence to a prescribed training regimen or expectancy effects. Furthermore, passive control groups are associated with higher effect size estimates [77] and can therefore lead to an overestimation of training effects. In contrast, comparison with an active control group offers greater differential conclusions regarding the effectiveness of the target training modality.

In the resistance training groups, the training intensity was individually adjusted using M-RM testing. However, in BT, training progression only occurred at the group level, as unstable equipment was changed from one phase to the next. On the one hand, this may have led to differences in effective training time between participants. While some were able to perform the exercises on the unstable materials, others had to get up and down more often due to uncertainty and loss of balance. On the other hand, since the internal training load generally determines the functional training outcome [118], considering and adjusting the individual training intensity could also be useful in BT in the future. However, assessing the training intensity of BT is considered challenging and lacking [111, 119, 120]. Future studies may prove that monitoring cognitive demand using subjective and (neuro-)physiological measures can help to tailor individual training progress [121, 122]. In addition, brain imaging techniques and molecular outcome measures could help elucidate the underlying mechanisms of the exercise-cognition interaction.

## Conclusion

In summary, the present study suggests that MRT leads to greater improvements in inhibitory control and processing speed compared to balance training. Future studies are required to determine whether these trends on performance level are also reflected in functional and structural changes in the brain. All in all, we demonstrated that MRT, as well as T-RT and BT, are suitable for improving physical and motor performance in relation to geriatric assessment and can effectively enhance cognitive performance. These findings support the role of physical exercise in the prevention and management of age-related physical and cognitive decline. It is important to note that recommendations focusing alone on resistance and balance training may overlook the potential added value of MRT as a multimodal training approach across the age spectrum. The challenge to postural control posed by MRT may stimulate cognition by requiring attentional resources that are not typically required in everyday life.

## Data Availability

The datasets used and/or analysed during the current study are available from the corresponding author on reasonable request.

## Abbreviations

ACC: Accuracy
BT: Balance Training
HSD: Honestly Significant Difference
M-RM: Multiple Repetition Maximum
MiniBESTest: Balance Evaluation System Test - short version
MoCA: Montreal Cognitive Assessment
MRT: Metastable Resistance Traing
T-RT: Traditional Resistance Training
RT: Reaction/Response Time
